# Seroprevalence and Risk Factors Associated with *Chlamydia abortus* Infection in Sheep and Goats in Eastern Saudi Arabia

**DOI:** 10.3390/pathogens10040489

**Published:** 2021-04-17

**Authors:** Mahmoud Fayez, Ahmed Elmoslemany, Mohammed Alorabi, Mohamed Alkafafy, Ibrahim Qasim, Theeb Al-Marri, Ibrahim Elsohaby

**Affiliations:** 1 Al-Ahsa Veterinary Diagnostic Lab, Ministry of Environment, Water and Agriculture, Al-Ahsa 31982, Saudi Arabia; mahmoudfayez30@hotmail.com (M.F.); theep8@hotmail.com (T.A.-M.); 2 Department of Bacteriology, Veterinary Serum and Vaccine Research Institute, Ministry of Agriculture, Cairo 131, Egypt; 3 Hygiene and Preventive Medicine Department, Faculty of Veterinary Medicine, Kafrelsheikh University, Kafr El-Sheikh 33516, Egypt; aelmoslemany@gmail.com; 4 Department of Biotechnology, College of Science, Taif University, P.O. Box 11099, Taif 21944, Saudi Arabia; maorabi@tu.edu.sa (M.A.); m.kafafy@tu.edu.sa (M.A.); 5 Department of Animal Resources, Ministry of Environment, Water and Agriculture, Riyadh 12629, Saudi Arabia; i.qasim@mewa.gov.sa; 6 Department of Animal Medicine, Faculty of Veterinary Medicine, Zagazig University, Zagazig City 44511, Egypt; 7 Department of Health Management, Atlantic Veterinary College, University of Prince Edward Island, Charlottetown, PE C1A 4P3, Canada

**Keywords:** *Chlamydia abortus*, seroprevalence, risk factors, multivariable analysis, sheep, goats, Saudi Arabia

## Abstract

*Chlamydia abortus* (*C. abortus*) is intracellular, Gram-negative bacterium that cause enzootic abortion in sheep and goats. Information on *C. abortus* seroprevalence and flock management risk factors associated with *C. abortus* seropositivity in sheep and goats in Saudi Arabia are scarce. The objectives of this study were to (i) estimate the animal, flock, and within-flock seroprevalence of *C. abortus* among Eastern Province sheep and goat flocks and (ii) identify the flock management and animal risk factors associated with *C. abortus* seropositivity in Eastern Province, Saudi Arabia. A cross-sectional study with a two-stage sampling process was carried out in the Eastern Province, Saudi Arabia, between 2015 and 2016. A total of 1717 sheep and 1101 goat serum samples were collected from 21 sheep and 14 goat flocks, then were tested for *C. abortus* antibodies using a commercial ELISA Kit. In addition, vaginal swabs and aborted tissue samples were collected from sheep (n = 48) and goats (n = 15) with recent history of abortion for detection of *C. abortus* *pmp* gene using PCR. A questionnaire was constructed to collect information about flock management and animal risk factors possibly associated with *C. abortus* infection in sheep and goats. The true sheep and goat-level seroprevalences were 11.1% (95% CI: 9.7–12.7) and 10.6% (95% CI: 8.8–12.5), respectively. The true flock-level seroprevalence was 100% for both sheep and goats. However, the average within sheep and goat flocks true seroprevalences were 9.6% (95% CI: 1.8–22.9) and 9.3% (95% CI: 1.8–19.5), respectively. Multivariable logistic regression revealed that introduction of new sheep to the flocks (OR = 2.6; 95% CI: 1.5–4.4), type of breeding system (OR = 1.8; 95% CI: 1.0–3.4), flocks allowing females in (OR = 1.9; 95% CI: 1.1–3.3) or females out (OR = 2.2; 95% CI: 1.1–4.3), and sheep age 1.4–2.8 years (OR = 1.9; 95% CI: 1.3–2.9) were potential risk factors for *C. abortus* seropositivity in sheep flocks. However, in goat flocks, the introduction of new goats to the flocks (OR: 1.9; 95% CI: 1.2–3.0) was identified as a risk factor, whereas good farm hygiene (OR: 0.3; 95% CI: 0.2–0.7) was identified as a protective factor. *C. abortus pmp* gene was identified in 45 (93.8%) and 15 (100%) of samples collected from sheep and goats, respectively. These results could be used to implement efficient management measures to prevent and control *C. abortus* infection in sheep and goats in Eastern Province, Saudi Arabia, but also could be used to reduce the risk of *C. abortus* infection in sheep and goat flocks with similar management practices in other regions.

## 1. Introduction

*Chlamydia* is an obligate intracellular, Gram-negative bacterium that causes various diseases in animals and humans [1,2]. However, *Chlamydia abortus* (*C. abortus*) and *Chlamydia pecorum* (*C. pecorum*) are the two *Chlamydia* species that cause serious infection in sheep and goats [3,4]. *C. abortus* is one of the main causes of abortion in sheep and goats worldwide [5,6] and usually transmitted through ingestion or inhalation of dust, food, and/or water contaminated with aborted animal uterine discharge, placenta, and fetus [3,7]. *C. abortus* cause enzootic abortion disease in sheep and goats which characterized clinically by abortion in the last 2–3 weeks of pregnancy [3]. Furthermore, *C. abortus* infection may cause stillbirth, premature lambing, and delivery of weak lamb/kids that generally die immediately [3,8]. In human, *C. abortus* infection is considered a zoonotic occupational disease and most of the infections were reported in veterinarians, livestock breeders, butchers, abattoir, and farm workers [3,9]. In humans, common symptoms in men and women are characterized by mild influenza-like illness or urethritis, however in severe cases, pregnant women are also at risk of abortion [10,11,12,13].

Several studies have investigated the seroprevalence of *C. abortus* in sheep and goats [14,15,16]. However, limited studies have assessed the flock-level risk factors associated with *C. abortus* seropositivity in sheep and goat flocks, including flock size, reproductive practices, husbandry, and farm sanitation [17,18,19]. Previous studies have reported that the real drivers of *C. abortus* infection in sheep [16,17], goats [19,20], camels [21], and cattle [22] are intrinsic farm factors such as production system and management practices, but a clearer understanding of these factors is needed. Knowledge of flock management practices and factors associated with the risk of *C. abortus* infection would prevent transmission and improve control strategies. In addition to the quality and effectiveness of the *C. abortus* diagnostic tests, *C. abortus* diagnosis requires either detection of bacteria/antigen by microscopic, immunofluorescence, ELISA, detection of the bacterial DNA by PCR and microarray [23,24], or detection of antibodies against *C. abortus* by ELISA [24,25].

In Saudi Arabia, few seroprevalence surveys have been conducted across different ruminants, including camels [26,27], sheep, and goats [28]. However, data on farm management risk factors for *C. abortus* infection in sheep and goats in Saudi Arabia is limited. Determination of *C. abortus* seroprevalence and risk factors associated with infection could help in the control of *C. abortus* in both animals and humans in Saudi Arabia. Therefore, the objectives of the present study were to (i) estimate the animal, flock, and within-flock seroprevalence of *C. abortus* among sheep and goat flocks in Eastern Province and (ii) identify the flock management and animal risk factors associated with *C. abortus* seroprevalence among sheep and goat flocks in Eastern Province, Saudi Arabia.

## 2. Results

### 2.1. Seroprevalence Animal- and Flock-Level

**Figure 1:**
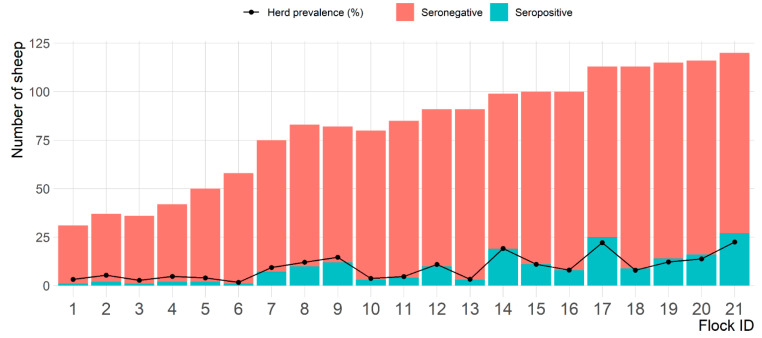
Number of seropositive and seronegative sheep and percentage of *Chlamydia abortus* seropositive sheep per flock on 21 sheep flocks in Eastern Province, Saudi Arabia.

Out of the 1717 sheep tested in the present study, 187 were seropositive to *C. abortus* with apparent individual sheep-level seroprevalence of 10.9% (95% CI: 9.5–12.5). At the flock-level, all 21 sheep flocks had at least one *C. abortus* seropositive sheep, giving an apparent sheep flock-level seroprevalence of 100%. However, the average of within-flock seroprevalence was 9.4% and ranged from 1.7% to 22.5% (Figure 1).

**Figure 2:**
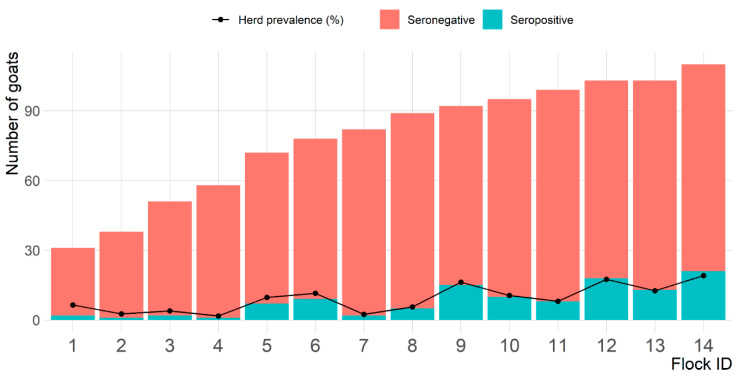
Number of seropositive and seronegative goats and percentage of *Chlamydia abortus* seropositive goats per flock on 14 goat flocks in Eastern Province, Saudi Arabia.

For goats, 114 were seropositive to *C. abortus* out of 1101 goats tested, with an apparent seroprevalence on the goat-level of 10.4% (95% CI: 8.6–12.3). Whereas at the flock level, all 14 goat flocks had at least one goat seropositive to *C. abortus* with an apparent goat flock-level seroprevalence of 100% and the average of within-flock seroprevalence of 9.1% and ranged from 1.7% to 19.1% (Figure 2).

The apparent animal, flock, and within-flock seroprevalences were adjusted to the ELISA test Se (98.1%) and Sp (100%). Thus, the true sheep and goat-level seroprevalences were 11.1% (95% CI: 9.7–12.7) and 10.6% (95% CI: 8.8–12.5), respectively. For both sheep and goats, the true flock-level seroprevalence was 100%, with an overall HSe and HSp were equal to 1.00 and 1.00, respectively. The average within sheep and goat flocks true seroprevalence were 9.58% (95% CI: 1.8–22.9) and 9.3 (95% CI: 1.8–19.5), respectively.

### 2.2. Risk Factor Analysis

The flock and animal risk factors associated with sheep and goats seropositivity in a univariable analysis at *P* < 0.20 are presented in Table 1 and Table 2, respectively. In sheep flocks, results showed a significant positive association (risk factor) between *C. abortus* seropositivity and larger flock size (*P* = 0.075), season (*P* = 0.003), recent history of abortion (*P* = 0.000), the introduction of new sheep to flock (*P* = 0.000), history of previous treatment (*P* = 0.001), type of breeding system (*P* = 0.000), sheep exchange during breeding (*P* = 0.007), and sheep age (*P* = 0.003). Whereas, farm hygiene (*P* = 0.002) was negatively associated (protective factors) with *C. abortus* seropositivity (Table 1). Similar results were found in goat flocks, except recent history of abortion (*P* = 0.969), the history of previous treatment (*P* = 0.654), and type of breeding system (*P* = 0.855) were not significantly associated with *C. abortus* seropositivity (Table 2).

Table 3 showed flock and animal level factors associated with sheep and goats seropositivity in a multivariable analysis at *P* < 0.05. In both sheep and goat populations, the flock size variable could be a potential confounder based on the change in the β-coefficient (>20%). Flock size was kept as a continuous variable in the sheep model, given that assumption of linearity was respected. However, this variable was kept as a categorized variable in the goat model, given linearity was not respected.

For sheep flocks, four factors were retained as being significantly associated with *C. abortus* seropositivity in the final multivariable logistic regression model. In addition to the flock size which correlated with introduction of new animals to the flock (0.77), open breeding system (0.78), and lower hygienic scores (−0.38). Results showed that farms that introduced new sheep in their flocks had significantly higher odds (OR = 2.6; 95% CI: 1.5–4.4) of being seropositive than farms that did not purchase any animals. The odds of being *C. abortus* seropositive is two times (OR = 1.8; 95% CI: 1.0–3.4) higher in open flocks than closed ones. Additionally, flocks allowing females in (OR = 1.9; 95% CI: 1.1–3.3) or females out (OR = 2.2; 95% CI: 1.1–4.3) were at higher risk of *C. abortus* seropositivity compared to flocks did not allow female in or out. On the other hand, sheep age 1.4–2.8 years had a higher risk (OR = 1.9; 95% CI: 1.3–2.9) than younger ones (<1.4 years). The Pearson’s chi-square (*P* = 0.75) and the Hosmer–Lemeshow (*P* = 0.80) fit statistics suggested a reasonable fit of the model.

For goat flocks, two factors were retained as being significantly associated with *C. abortus* seropositivity in the final multivariable logistic regression model. The odds of *C. abortus* seropositivity were higher for flocks introducing/purchased new goats (OR = 1.9; 95% CI: 1.2–3.0) than flocks that did not introduce new animals to flocks. However, results showed that flocks with bad hygienic measures were three times (OR = 3.0; 95% CI: 1.5–6.0) more likely to be seropositive for *C. abortus* compared to flocks with good hygienic measures. The Pearson’s chi-square (*P* = 0.4) and the Hosmer–Lemeshow (*P* = 0.9) fit statistics suggested a reasonable fit of the model.

### 2.3. Molecular Identification of C. abortus

Vaginal swabs and aborted tissue samples were collected from 17 (81%) and 11 (78.6%) out of 21 and 14 sheep and goat flocks, respectively. *C. abortus pmp* gene was identified in 45 (93.8%) and 15 (100%) of samples collected from sheep and goats, respectively (Table 4).

## 3. Discussion

### 3.1. Seroprevalence of C. abortus

In this study, the true seroprevalence of *C. abortus* antibodies at sheep-level was 11.1%, which was higher than the 7.5% reported previously in sheep in Riyadh, Saudi Arabia; similar to the 10.6% reported in sheep in India; but lower than the 40.1% and 24.5% reported in sheep in China and Algeria, respectively. The true seroprevalence at goat-level in this study was 10.4%, which was similar to the 11.4% reported in Jordan and lower than the 34.5% reported in Saudi Arabia and 33% reported in goats in Spain [19].

Our results revealed that 100% of the sheep and goat flocks had at least one *C. abortus* seropositive. This flock-level seroprevalence was similar to the 100% reported in both sheep and goat flocks in Jordan but was higher than the 70.4% of sheep flocks in Algeria and 78.6% of goat flocks in China [29]. The average within sheep and goat flock seroprevalences in this study were 9.6% (ranging: 1.8 to 22.9) and 9.3% (ranging: 1.8 to 19.5), respectively, indicating the spread of *C. abortus* infection within sheep and goat flocks in Eastern Saudi Arabia. This result was similar to the range of within flock seroprevalence 3.7% to 25.0% reported in sheep in Costa Rica [30] and the 0.0% to 29.9% reported in goats in China [29]. Such variation in *C. abortus* seroprevalence is likely due to differences in sheep and goat breeds, husbandry practices, sanitation, time of sampling, and serological test used. In addition, other reasons for the variations of seroprevalence may be related to differences in climate conditions, including temperature, rainfall, and altitude [31]. However, this study showed no significant differences in the seroprevalence of *C. abortus* antibodies between sheep and goats at the animal- and flock-level. This may attribute to the pasture restriction in the study area, which allows sheep and goat flocks to graze in a common pasture. Previous studies have reported that grazing on contaminated pasture is a source of *C. abortus* transmission among animals [6,32].

In Saudi Arabia, vaccination against chlamydiosis is not applicable. Thus, the relatively high seroprevalence of *C. abortus* in the current study is suspected and could be explained by the fact that the study area (Eastern Province) has borders with five *C. abortus* endemic countries [33,34,35]. Furthermore, sheep and goat flocks are seminomadic and moving for long distances every day during the grazing season, which might contribute to the horizontal spread of *C. abortus* to a large number of flocks.

### 3.2. Risk Factors

Various risk factors were assessed to be associated with the seroprevalence of *C. abortus* in sheep and goat flocks in Saudi Arabia. In the present study, the highest sheep flock (22.9%) and goat flock (19.5%) seroprevalence was reported in large size flocks (>200), which was confirmed by the univariable analysis. This finding is consistent with previous studies from Tunisia and Spain, which indicated that larger size flocks are associated with higher *C. abortus* seropositivity compared to smaller size flocks. In contrast, other studies from Iran, Algeria, and Brazil [17] have reported no association between flock size and *C. abortus* seropositivity. The possible explanation that sheep/goats overcrowding in large size flocks may influence animal welfare and hygienic measures, which increase the risk of *C. abortus* transmission [4,6]. In addition, the association between a higher *C. abortus* seropositivity and the increased flock size may also be related to the larger number of visitors to the farms by veterinarians, feed suppliers, and farm workers [14,36]. In this study, larger size flocks were correlated with introduction of new animals to the flock (0.77), open breeding system (0.78), and lower hygienic scores (−0.38).

Climates play a role in *C. abortus* infection [31]. The warm and humid conditions are favorable for the survival of *Chlamydia* [37]. In the present study, the seroprevalence of *C. abortus* in winter was significantly higher in sheep and goat flocks than in summer. This result may be due to the climate of Eastern Province is desert which mainly dried hot in summer and mild humid in winter. Similarly, previous studies have reported that season/climate is a risk factor for *C. abortus* infection in sheep [38] and goat [29] flocks in China.

Several studies have pointed out that the seroprevalence of *C. abortus* in small ruminants varies based on the management system operated on the flock [19,39,40]. Therefore, we could assume that the flock management practices in Saudi Arabia play a significant role in the *C. abortus* seroprevalence reported in the present study. The univariable risk factor analysis of the flock management revealed several factors to be significantly associated with *C. abortus* seroprevalence. In sheep flocks, results revealed that flocks with a recent history of abortion, history of treatment, introduced new sheep to the flock, exchanged rams during breeding were identified as risk factors for *C. abortus* infection. However, in goat flocks, only the goat age, introduction of new goats to flock, and goat exchange during breeding were identified as risk factors for *C. abortus* infection. Furthermore, it was revealed that the seroprevalence of *C. abortus* in the present study significantly decreases in sheep and goat flocks, where good hygienic measures were applied. Similar results were previously reported in sheep flocks in China [38], Egypt, Algeria, Brazil, Costa Rican, and goat flocks in China [29]. In contrast, a recent study in Tunisian sheep flocks has reported no association between seroprevalence of *C. abortus* and the number of ewes with abortion history, the exchange of breeding male, and the frequency of disinfection [15].

The risk of sheep and goat flocks acquiring *C. abortus* infection seems to be complex. The multivariable analysis in the present study confirmed that four different variables were associated with *C. abortus* seropositivity in sheep flocks. The multivariable model showed that flocks using open breeding system, adding new sheep and exchanging sheep during breeding are at higher risk of *C. abortus* seropositivity. Similar to our results, Mamlouk et al. reported that the absence of previous control of sheep, when introduced to the flock, is a risk for *C. abortus* seropositivity. Furthermore, Barkallah et al. observed that quarantine had a significant relation with *C. abortus* infection in sheep. The observed high seroprevalence of *C. abortus* in older sheep in this study was in agreement with previous studies, which reported an association between the increase of *C. abortus* seroprevalence and sheep age [41,42]. This result may be due to earlier infection or the high chance of exposure to the source of infection with age increase [38].

In goat flocks, results showed a significant relationship between *C. abortus* seropositivity and the introduction of new goats to the flock. Some farms purchased new goats without pre-testing and history taking, leading to an increased risk of infection. This result is consistent with Santos et al. [17], who reported that sharing goats for breeding between farms contributed to the spread of *C. abortus* infection from one flock to another. On the other hand, the risk of *C. abortus* seropositivity in goat flocks with good hygienic measures is lower than flocks with bad hygienic measures, which contrasts with Santos et al., who reported no association between use of disinfectant and risk of *C. abortus* seropositivity.

### 3.3. Study Limitations

The ELISA test was used in the present study to assess the seroprevalence of *C. abortus* antibodies in sheep and goat serum samples. Although the sensitivity and specificity of the used ELISA are high but indeed not 100%, so some misclassification errors may therefore occur, which may add some noise to the observed relationship between dependent and independent variables. Furthermore, the antigenic cross-reactivity between *C. abortus* and *C. pecorum* in sheep may result in an overestimation of seroprevalence [43,44]. Because of the relatively small number of goat flocks included in the study, only variables that were strongly associated with *C. abortus* could be evaluated in the final model. The absence of a particular variable from the final model may be due to the limited sample size. However, the identification of at least one PCR positive animal in each flock decreases the impact of these limitations on this study results.

## 4. Materials and Methods

### 4.1. Study Area

**Figure 3:**
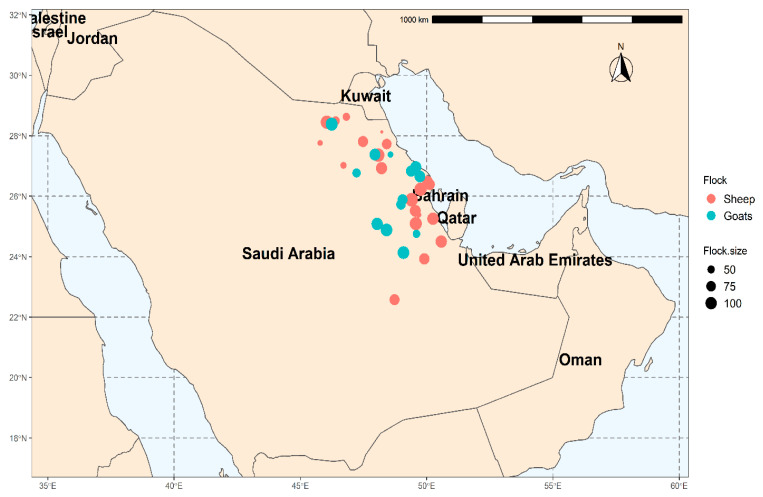
Map of Saudi Arabia showed the locations of sampled sheep and goat flocks.

Eastern Province (22°30′ N, 51°00′ E) is located in the eastern side of Saudi Arabia, at 390 km from the capital Riyadh and shares the borders with five countries, including Oman, United Arab Emirates, Qatar, Kuwait, and Iraq (Figure 3). The Eastern Province is the third most populated province in Saudi Arabia, with diverse climatic conditions ranging from semi-desert to desert. According to the General Authority for Statistics for 2015, sheep and goat populations in Eastern Province represent 13% and 2.5% of the total sheep and goat populations in the kingdom.

The production system of small ruminants is mainly of the seminomadic type as sheep and goats move out from their raising farms for grazing during early March until August when pastures are mostly available. During the fall and winter months, sheep and goats are housed and fed on concentrates. Sheep and goats in Saudi Arabia are mainly raised for meat production.

### 4.2. Study Design

A cross-sectional study with a two-stage sampling process was carried during 2015-2016. The total number of sheep and goat flocks to be sampled was determined using the ‘*epi.ssclus2estb*’ function of the ‘epiR’ package with 50% an expected seroprevalence, 5% absolute precision, and 95% confidence interval (CI). The required number of flocks was determined to be 12 flocks. For the sheep population, the number of flocks was increased to 21, and for the goat population increased to 14 flocks to improve the precision of the estimated seroprevalence in sheep and goats [45]. Selected sheep flocks size ranged from 40 to 800 (median = 350) sheep per flock. However, selected goat flocks size ranged from 40 to 500 (median = 290) goats per flock. The number of sheep and goats sampled per flock was calculated according to Cannon and Roe, using an expected seroprevalence of 8% with a 95% CI to detect at least one seropositive.

### 4.3. Sample and Data Collection

A two-stage random sampling process was carried out. Flocks were selected in the first stage and animals within flocks were selected in the second stage. In total, blood samples were collected from 1717 sheep in 21 flocks and 1101 goats in 14 flocks, with an average of 80 animals sampled per flock. In addition, vaginal swabs and aborted tissue samples were collected from a number of sheep (n = 48) and goats (n = 15) with recent history of abortion. All samples were labeled with flock and animal ID and sampling dates and then transported to the laboratory for serological analysis.

A questionnaire was constructed to collect information about flock management and animal risk factors possibly associated with *C. abortus* infection in sheep and goats. The questionnaire consisted of 11 closed and open-ended questions written in Arabic and available from the corresponding author upon request. The questionnaire was completed by face-to-face interviews with the flock owner on the day of sampling. Questions covered the flock characteristics and management practices, including flock size (small (≤200 animal/flock) and large (>200 animal/flock)), the season of sampling, the recent history of abortion (Yes and No), the introduction of new animals to flock (Yes and No), mixed breeding (Yes and No), farm hygiene (Bad and Good), history of previous treatment (Yes and No), type of breeding system (Open and Closed), animal exchange during breeding (No, Female in and out), and type of the used vaccine. In addition to the animal age, sex, and breed, this information is presented in Supplementary Table S1.

### 4.4. Serological Examination

Serum samples were tested for antibodies against *C. abortus* infection using commercial indirect ELISA kits (IDEXX Chlamydiosis Total Ab Test, IDEXX Laboratories, Broomfield, CO, USA) according to manufacture instructions. Briefly, serum samples and controls were diluted at 1:400 and then tested in duplicates. Microplate ELISA reader measured the optical densities (OD) at 450 nm. The average OD was estimated for duplicate samples and controls, then used to calculate the OD% by the following equation:

OD (%) = (OD _sample_ − OD _negative control_)/(OD _positive control_ − OD _negative control_) × 100. (1)

Any sample with OD% >40% was considered seropositive; if the OD% was between ≥30 and ≤40, the result was considered doubtful, while any sample with an OD% <30% was classified as seronegative. Regarding the diagnostic performance of the IDEXX ELISA’s kites used in this study, Wilson et al. [46] evaluated the accuracy of ELISA in the absence of a gold standard and reported a 98.1% sensitivity (Se) and 100% specificity (Sp) in sheep and goats.

### 4.5. Molecular Identification of C. abortus

DNA was extracted from vaginal swabs and aborted tissue samples using the Qiagen QIAamp DNA mini kit (Qiagen, Courtaboeuf, France) according to the manufacturer’s instructions. Purified DNA was amplified by PCR for detection of the *pmp* gene of *C. abortus* using the primer (CpsiA (5′-ATGAAACATCCAGTCTACTGG-3′) and CpsiB (5′-TTGTGTAGTAATATTATCAAA-3′)) previously described by Greco et al. [47]. Briefly, 2 μL sample of each purified genomic DNAs was amplified in 20 μL of the final volume of a 2X HotStartTaq Plus Master Mix (QIAGEN, Germantown, MD, USA) containing 1.5 mM MgCl2, 200 μM of each dNTP,1-unit HotStartTaq Plus DNA polymerase, and 10 μM of each forward and reverse primers. The PCR assay was performed using DNA thermal cycler I (BIORAD, CA, USA) according to Greco et al. [47]. The amplified PCR products were electrophoresed in 1.5% agarose gel stained with ethidium bromide and documented using an ultraviolet gel documentation system (BIO-RAD, Hercules, CA, USA).

### 4.6. Statistical Analysis

Epidemiological data and results of the serological analysis were introduced into Stata Statistical Software v. 15 (Stata Corp, College Station, TX, USA) and R software (R Core Team, 2019; version 3.5.3) for descriptive and statistical data analysis with results considered significant at *P-value* < 0.05. The apparent seroprevalence of *C. abortus* at the individual animal-level was estimated as the ratio of seropositive sheep/goat to the total number of sheep/goats examined. However, the true seroprevalence of *C. abortus* at the animal-level was estimated according to Thrusfield [48] using the ‘*epi.prv*’ function from the ‘epiR’ package in R [49]. The flock-level seroprevalence was estimated from the ratio of positive flocks to the total number of flocks tested. Flocks that contain at least one seropositive sheep/goat were considered positive. The flock seroprevalence was adjusted for Se and Sp of ELISA test used in the study to obtain the true flock seroprevalence using the following formula [50]:

THP = (AHP + HSp − 1)/(HSe + HSp − 1),(2)

where THP and AHP are the true and apparent flock seroprevalence, respectively. HSe and HSp refer to the flock-test sensitivity and specificity, respectively, based on animal-test results, calculated using ‘*epi.herdtest*’ function from Package ‘epiR’.

Analysis of the risk factors potentially associated with *C. abortus* seropositivity was evaluated in two steps, using univariable and multivariable logistic regression models. Firstly, univariable screening of all risk factors for association with *C. abortus* seropositivity at liberal *P-value* < 0.20 using unconditional associations. Significant variables in the univariable analysis were checked for collinearity using Spearman correlation coefficients and were considered collinear when coefficient >0.8 [51]. Retained variables were used to conduct multivariable analysis, and non-significant variables were removed sequentially using backward elimination at *P*-*value* < 0.05. The fit of the final model was evaluated using Hosmer–Lemeshow goodness-of-fit statistics [52].

## 5. Conclusions

The present study revealed that *C. abortus* seroprevalence in sheep (11.1%) and goats (10.4%) is relatively high in Eastern Province, Saudi Arabia. Consequently, some management practices must be implemented, especially the risk factors identified in this work, such as introducing new animals to the flock and improving farm hygienic measures. Since *C. abortus* is a zoonotic disease that can cause health and economic loss in humans and livestock, thus the use of a chlamydial vaccine is strongly recommended. In addition to the implementation of integrated control and efficient management measures to prevent and control *C. abortus* infection in sheep and goats in Eastern Province, Saudi Arabia, it also could be used to reduce the risk of *C. abortus* infection in sheep and goat flocks with similar management practices in other regions.

## Figures and Tables

**Table 1:** Univariable analysis of flock and animal risk factors association (*P* < 0.20) with *Chlamydia abortus* seropositivity among sheep sampled from 21 flocks in Eastern Province, Saudi Arabia.

Factors	Frequency of Examined Sheep (%)	Prevalence of Seropositive Sheep (%)	OR (95% CI) ^1^	*P*-Value
		Flock Level		
Flock size				
Small (≤200)	28.8	7.7	1.0 (ref.)	
Large (>200)	71.2	12.2	1.8 (0.9–3.4)	0.075
Season				
Summer	18.7	6.5	1.0 (ref.)	0.003
Winter	41.4	15.0	2.7 (1.3–5.5)	0.006
Spring	23.3	11.3	1.8 (0.8–3.9)	0.131
Autumn	16.6	5.3	0.8 (0.3–2.1)	0.710
Recent history of abortion				
No	85.6	6.9	1.0 (ref.)	
Yes	14.4	34.8	308.3 (91.0–1030.6)	0.000
Introductions of new sheep to the flock				
No	61.4	7.1	1.0 (ref.)	
Yes	38.6	16.9	2.7 (1.8–4.2)	0.000
Mixed breeding farm				
No	82.8	11.1	1.0 (ref.)	
Yes	17.2	10.1	1.2 (0.5–2.8)	0.713
Farm hygiene				
Bad	85.9	12.1	1.0 (ref.)	
Good	14.1	3.3	0.3 (0.1–0.6)	0.002
History of previous treatment				
No	85.5	10.0	1.0 (ref.)	
Yes	14.5	16.1	1.9 (1.3–2.9)	0.001
Type of breeding system				
Closed	49.5	6.9	1.0 (ref.)	
Open	50.5	14.8	2.5 (1.5–4.0)	0.000
Sheep exchange during breeding				
No	33.0	6.4	1.0 (ref.)	0.007
Female out	17.2	10.9	2.2 (1.0–5.0)	0.055
Female in	49.8	13.9	2.7 (1.4–5.0)	0.002
Vaccine type				
Clostridia (C) only	14.0	8.3	1.0 (ref.)	0.523
Pasteurella (P) only	9.2	5.7	0.6 (0.2–2.4)	0.494
C + P	34.3	12.1	1.4 (0.5–3.6)	0.500
C + P + PPR	42.5	11.9	1.4 (0.6–3.4)	0.501
		Animal level		
Age				
<1.4 year	26.1	7.2	1.0 (ref.)	0.003
1.4–2.8 years	51.0	13.4	2.0 (1.3–3.0)	0.002
≥ 2.8 years	22.9	9.7	1.3 (0.8–2.2)	0.267
Sex				
Male	7.4	12.6	1.0 (ref.)	
Female	92.6	10.8	0.8 (0.5–1.5)	0.506
Breed				
Awassi (Naeimi)	40.1	12.5	1.0 (ref.)	0.352
Najdi	26.3	9.1	0.7 (0.4–1.5)	0.421
Sawakin	10.5	6.2	0.5 (0.2–1.4)	0.182
Mixed	23.1	12.4	1.2 (0.6–2.7)	0.598

^1^ OR: odds ratio; CI: confidence interval.

**Table 2:** Univariable analysis of flock and animal risk factors association (*P* < 0.20) with *Chlamydia abortus* seropositivity among goats sampled from 14 flocks in Eastern Province, Saudi Arabia.

Factors	Frequency of Examined Goats (%)	Prevalence of Seropositive Goats (%)	OR (95% CI) ^1^	*P*-Value
		Flock Level		
Flock size				
Small (≤200)	29.8	6.7	1.0 (ref.)	
Large (>200)	70.2	11.9	2.0 (1.0–4.0)	0.067
Season				
Summer	12.7	2.1	1.0 (ref.)	0.038
Winter	47.6	12.2	6.2 (1.7–22.4)	0.005
Spring	21.4	12.3	6.0 (1.6–23.1)	0.009
Autumn	18.3	9.0	4.5 (1.1–17.4)	0.032
Recent history of abortion				
No	97.7	8.2	1.0 (ref.)	
Yes	2.3	100	644.7	0.969
Introductions of new goats to the flock				
No	80.7	8.5	1.0 (ref.)	
Yes	19.4	18.3	2.6 (1.4–5.0)	0.004
Mixed breeding farm				
No	74.3	11.1	1.0 (ref.)	
Yes	25.7	8.1	0.8 (0.4–2.0)	0.692
Farm hygiene				
Bad	69.9	12.9	1.0 (ref.)	
Good	30.2	4.5	0.3 (0.2–0.6)	0.000
History of previous treatment				
No	97.2	10.5	1.0 (ref.)	
Yes	2.8	6.5	0.7 (0.1–4.1)	0.654
Type of breeding system				
Closed	91.0	10.6	1.0 (ref.)	
Open	9.0	8.1	0.9 (0.2–3.4)	0.855
Goat exchange during breeding				
No	38.3	4.8	1.0 (ref.)	
Female out	26.3	14.8	3.5 (2.0–6.3)	0.000
Female in	35.4	13.1	3.0 (1.7–5.3)	0.000
Vaccine type				
Clostridia (C) only	12.1	3.0	1.0 (ref.)	0.109
Pasteurella (P) only	9.0	8.1	2.9 (0.6–13.1)	0.175
C + P	14.9	13.4	4.9 (1.3–18.4)	0.017
C + P + PPR	64.0	11.4	3.8 (1.2–12.3)	0.028
		Animal level		
Age				
≤2 year	51.2	9.2	1.0 (ref.)	
>2 year	48.8	11.6	1.4 (0.9–2.0)	0.121
Sex				
Male	16.0	10.3	1.0 (ref.)	
Female	84.0	10.8	1.0 (0.6–0.6)	0.879
Breed				
Aradi	41.7	13.1	1.0 (ref.)	0.255
Damascus	27.3	10.0	0.7 (0.3–1.5)	0.367
Mixed	31.0	7.0	0.5 (0.2–1.1)	0.105

^1^ OR: odds ratio; CI: confidence interval.

**Table 3:** Multivariable logistic regression analysis of flock and animal risk factors association (*P* < 0.05) with *Chlamydia abortus* seropositivity among sheep and goats sampled from flocks in Eastern Province, Saudi Arabia.

Factors	OR (95% CI) ^1^	*P-Value*
Sheep		
Flock size (continuous)	0.99 (0.99–1.0)	0.146
Introductions of new sheep to the flock		
No	1.0 (ref.)	
Yes	2.6 (1.5–4.4)	0.001
Type of breeding system		
Closed	1.0 (ref.)	
Open	1.8 (1.0–3.4)	0.056
Sheep exchange during breeding		
No	1.0 (ref.)	0.041
Female out	2.2 (1.1–4.3)	0.026
Female in	1.9 (1.1–3.3)	0.020
Age		
<1.4 years	1.0 (ref.)	0.004
1.4–2.8 years	1.9 (1.3–2.9)	0.022
≥ 2.8 years	1.3 (0.8–2.1)	0.313
Goats		
Flock size		
Small (≤200)	1.0 (ref.)	
Large (>200)	0.8 (0.4–1.5)	0.449
Introductions of new goats to the flock		
No	1.0 (ref.)	
Yes	1.9 (1.2–3.0)	0.004
Farm hygiene		
Bad	1.0 (ref.)	
Good	0.3 (0.2–0.7)	0.002

^1^ OR: odds ratio; CI: confidence interval.

**Table 4:** Number of *Chlamydia abortus* PCR positive vaginal swabs and aborted tissue samples collected from sheep and goats flocks with recent history of abortion.

No. of Sheep						No. of Goats					
Flock ID	Tested	Sero-Positive	Recent Abortion	Vaginal, Aborted Tissue Samples	PCR Positive	Flock ID	Tested	Sero-Positive	Recent Abortion	Vaginal, Aborted Tissue Samples	PCR Positive
1	31	1	0	0	0	1	31	2	0	0	0
2	37	2	1	1	1	2	38	1	0	0	0
3	36	1	0	0	0	3	51	2	0	0	0
4	42	2	2	2	2	4	58	1	1	1	1
5	50	2	2	2	1	5	72	7	2	1	1
6	58	1	0	0	0	6	78	9	3	2	2
7	75	7	75	3	2	7	82	2	1	1	1
8	83	10	5	3	2	8	89	5	1	1	1
9	82	12	7	4	4	9	92	15	3	2	2
10	80	3	0	0	0	10	95	10	2	1	1
11	85	4	1	1	1	11	99	8	1	1	1
12	91	10	2	2	2	12	103	18	3	1	1
13	91	3	1	1	1	13	103	13	4	2	2
14	99	19	9	4	4	14	110	21	5	2	2
15	100	11	5	3	3						
16	100	8	99	3	3						
17	113	25	10	6	6						
18	113	9	3	1	1						
19	115	14	8	5	5						
20	116	16	9	4	4						
21	120	27	8	3	3						
Total	1717	187	247	48	45	Total	1101	114	26	15	15

## Data Availability

The data presented in this study are available on request from the corresponding author.

## Supplementary Materials

The following are available online at https://www.mdpi.com/article/10.3390/pathogens10040489/s1.
