# Supplementary material for: Seroprevalence and Risk Factors Associated with Chlamydia abortus Infection in Sheep and Goats in Eastern Saudi Arabia
Source: Pathogens. 2021 Apr 17;10(4):489. doi: 10.3390/pathogens10040489 (PMC8072607; doi:10.3390/pathogens10040489)
Supplement: Supplementary file 1 [file pathogens-10-00489-s001.zip › pathogens-1166379-supplementary.pdf]

Table S1. Description and categories of the collected variables.

	Sheep			Goats		
**Factors**	**Categories**	**Frequency**	**%**	**Categories**	**Frequency**	**%**
**Age**						
	<1.4 year	448	26.1	≤2 year	564	51.2
	1.4 – 2.8 years	876	51.0	>2 year	537	48.8
	≥ 2.8 years	393	22.9			
**Sex**						
	Male	127	7.4	Male	176	16.0
	Female	1590	92.6	Female	925	84.0
**Breed**						
	Awassi (Naeimi)	689	40.1	Aradi	459	41.7
	Najdi	452	26.3	Damascus	301	27.3
	Sawakin	181	10.5	Mixed	341	31.0
	Mixed	395	23.1			
**Flock size**						
	Small (≤200)	494	28.8	Small (≤200)	328	29.8
	Large (>200)	1223	71.2	Large (>200)	773	70.2
**Season**						
	Summer	322	18.7	Summer	140	12.7
	Winter	710	41.4	Winter	524	47.6
	Spring	400	23.3	Spring	236	21.4
	Autumn	285	16.6	Autumn	201	18.3
**Recent history of abortion**						
	No	1470	85.6	No	1075	97.7
	Yes	247	14.4	Yes	26	2.3
**Introductions of new sheep to the flock**						
	No	1054	61.4	No	888	80.7
	Yes	663	38.6	Yes	213	19.4
**Mixed breeding farm**						
	No	1421	82.8	No	818	74.3
	Yes	296	17.2	Yes	283	25.7
**Farm hygiene**						
	Bad	1475	85.9	Bad	769	69.9
	Good	242	14.1	Good	332	30.2

**History of previous treatment**						
	No	1468	85.5	No	1070	97.2
	Yes	249	14.5	Yes	31	2.8
**Type of breeding system**						
	Closed	850	49.5	Closed	1002	91.0
	Open	867	50.5	Open	99	9.0
**Sheep exchange during breeding**						
	No	567	33.0	No	421	38.3
	Female out	295	17.2	Female out	290	26.3
	Female in	855	49.8	Female in	390	35.4
**Vaccine type**						
	Clostridia (C) only	240	14.0	Clostridia (C) only	133	12.1
	Pasteurella (P) only	158	9.2	Pasteurella (P) only	99	9.0
	C + P	589	34.3	C + P	164	14.9
	C + P + PPR	730	42.5	C + P + PPR	705	64.0
**Total**		**1717**			**1101**	
